# Mental health and cognition in relation to adherence to antiretroviral therapy among people living with HIV in Kazakhstan: a cross‐sectional study

**DOI:** 10.1002/jia2.26320

**Published:** 2024-07-18

**Authors:** Gaukhar Mergenova, Alissa Davis, Louisa Gilbert, Nabila El‐Bassel, Assel Terlikbayeva, Sholpan Primbetova, Zhamilya Nugmanova, Andrea Norcini Pala, Deborah Gustafson, Susan L. Rosenthal, Alfiya Y. Denebayeva, Jack DeHovitz

**Affiliations:** ^1^ Global Health Research Center of Central Asia Almaty Kazakhstan; ^2^ Asfendiyarov Kazakh National Medical University Almaty Kazakhstan; ^3^ Columbia University School of Social Work Columbia University New York City New York USA; ^4^ State University of New York Downstate Health Sciences University Brooklyn New York USA; ^5^ Department of Pediatrics and Psychiatry Vagelos College of Physicians and Surgeons Columbia University Irving Medical Center New York City New York USA; ^6^ Almaty City AIDS Center Almaty Kazakhstan

**Keywords:** HIV, ART adherence, depression, anxiety, cognition, forgetfulness

## Abstract

**Introduction:**

There is a research gap in how mental health and cognition are associated with antiretroviral treatment (ART) adherence among people living with HIV (PLWH) in Kazakhstan.

**Methods:**

We randomly selected and enrolled 230 PLWH from the Almaty City AIDS Center registry (June−November 2019) into a cross‐sectional study. We examined associations between self‐reported ART adherence for the last 1 and 2 weeks; the Adherence Self‐Efficacy Scale (ASES) and symptoms of depression (Patient Health Questionnaire‐9 [PHQ‐9]), anxiety (Generalized Anxiety Disorder tool [GAD‐7]), post‐traumatic stress disorder (PTSD Checklist [PTSD]); cognitive function (PROMIS v2.0 Adult Cognitive Function 8a short form) and forgetfulness (Forgetfulness Assessment Inventory). We used cut points of ≥5 for at least mild and ≥10 for at least moderate symptom severity for PHQ‐9 and GAD‐7 and of ≥44 for PTSD. Logistic and linear regression analyses were used.

**Results:**

Participants’ median age was 40.0 (IQR: 34−47) with 40.9% (*n* = 94) of females in the sample. Those who missed at least one pill for the last 2 weeks had higher odds of reporting at least mild depression (aOR = 3.34, 95% CI: 1.22–9.11, *p* < 0.05); mild anxiety (aOR = 3.27, 95% CI: 1.20–8.92, *p* < 0.05); and PTSD (aOR = 4.04, 95% CI: 1.15–14.21, *p* < 0.05) symptoms. Participants who missed at least one pill for the last week had higher odds of at least mild depression (aOR = 7.74, 95% CI: 1.31–45.7, *p* < 0.05), moderate anxiety (aOR = 21.33, 95% CI: 3.24–140.33, *p* < 0.005) and PTSD (aOR = 13.81, 95% CI: 2.36–80.84, *p* < 0.005) symptoms. Participants with better cognitive function had lower odds of non‐adherence over the last week (aOR = 0.88, 95% CI: 0.81–0.96, *p* < 0.005) and higher ASES scores (β = 0.26, 95% CI: 0.13–0.40, *p* < 0.005). Poor memory was associated with higher odds of non‐adherence over the last week (aOR = 4.64, 95% CI: 1.76–12.24, *p* < 0.005) and lower ASES score (β = −0.31, 95% CI: −0.45 to 0.16, *p* < 0.005). Those who had at least mild depression (β = −0.21, 95% CI: −0.35 to −0.07, *p* < 0.005); moderate anxiety (β = −0.21, 95% CI: −0.34 to −0.07, *p* < 0.005) and PTSD (β = −0.19, 95% CI: −0.33 to −0.05, *p* < 0.005) symptoms had lower ASES scores.

**Conclusions:**

Depression, anxiety and PTSD symptoms, poorer cognition, and forgetfulness were associated with poorer ART adherence and worse adherence self‐efficacy. It is crucial to assess and treat mental illness and provide support for PLWH with worsened cognition to enhance ART adherence.

## 1 INTRODUCTION

Eastern Europe and central Asia (EECA), including Kazakhstan [1], is the only region with a rising HIV incidence, with an estimated 33,000 people living with HIV (PLWH). Of these, 80% knew their HIV status, 64% received antiretroviral treatment (ART) and 55% had a viral load of <1000 copies/ml [2].

In 2019, an estimated 13% of the global population was living with a mood disorder (depression 29%, anxiety 31%), and 82% in low‐ and middle‐income countries [3]. Among PLWH globally, the prevalence of mental health disorders is high [4, 5, 6, 7, 8, 9, 10, 11, 12, 13, 14, 15, 16, 17, 18, 19, 20]. Prevalence of depression among PLWH from high‐income countries varied between 5% and 20% [9, 21, 22]. One study reported that ∼10% of PLWH had symptoms of depression in Almaty, Kazakhstan [23]. Multiple studies revealed associations between mental health disorders and high viral load, which are often associated with poor adherence [7, 8, 24–30]. HIV affects cognitive function and mental health [31, 32, 33, 34, 35, 36, 37]. Also, some ART regimens may have neuropsychiatric side effects [38].

Published literature indicates that ART adherence is associated with mental health and cognitive function among PLWH globally. However, there is limited data on associations of ART adherence and ART self‐efficacy with mental health symptoms and cognition among PLWH in Kazakhstan. We hypothesized that symptoms of depression, anxiety and post‐traumatic stress disorder (PTSD) as well as poorer cognition, are associated with poorer ART adherence and lower ART self‐efficacy. In this cross‐sectional study, we examined the association of ART adherence and ART self‐efficacy with depression symptoms, anxiety symptoms, clinically relevant PTSD and cognitive function among PLWH in Almaty, Kazakhstan.

## 2 METHODS

This cross‐sectional study took place in Almaty, Kazakhstan. In Kazakhstan, all HIV‐related services (testing, treatment and prevention) are provided by AIDS centres. The Almaty AIDS City Center is the only healthcare facility that provides HIV‐related services for PLWH in Almaty. Eligibility criteria were: (1) 18 years old or older; and (2) prescribed ART for at least 6 months. PLWH were excluded if they (1) showed evidence of very severe psychiatric or cognitive impairment or (2) were not fluent in Russian. We randomly selected 246 participants from the Almaty AIDS City Center roster and 230 were successfully enrolled in the study between June and November 2019. Each participant signed a consent form and received compensation (∼USD$10) for a 90‐minute computerized self‐assisted interview. The interview was monitored by a trained psychologist, who also provided a consultation and referral for treatment. A nurse extracted clinical data from the medical records of participants. We were unable to utilize CD4^+^ cell count and HIV viral load because laboratory tests were collected across wide time intervals (every 6 months), which did not align with survey dates. All data were collected using surveys.

### 2.1 Ethical approvals

The study obtained ethical approvals from the Al‐Farabi Kazakh National University Ethics Committee and the Columbia University Institutional Review Board.

### 2.2 Measures

#### 2.2.1 Dependent variables

##### 2.2.1.1 Self‐reported ART adherence

Self‐reported ART adherence was determined by answering the question: “When was the last time you missed taking any of your antiretroviral medications?” Response options were: “last 3 days,” “within the past week,” “1−2 weeks ago,” “2−4 weeks ago,” “3 months ago,” “more than 3 months ago,” “never skip medications.”

Adults who reported (1) missing at least one pill in the last week and (2) missing at least one pill in the last 2 weeks were defined as two categories of ART non‐adherent participants, while those who did not miss any pills in these time periods were defined as ART adherent.

##### 2.2.1.2 Adherence self‐efficacy

The 12‐item Adherence Self‐Efficacy Scale (ASES) was used to assess PLWH's adherence self‐efficacy [39]. Responses range from 1 (cannot do it at all) to 10 (certainly can do it). Item scores were averaged for each respondent, with higher scores indicating higher adherence self‐efficacy [39]. The overall score ranged from 0 to 120. Cronbach's alpha in our sample was α = 0.97.

##### 2.2.1.3 Independent variables

Socio‐demographic variables included age, gender, employment status, marital status, ethnicity, history of ever being diagnosed with infectious diseases, self‐reported history of ever being diagnosed with any mental illness and education level. Substance use variables included injection drug use ever in a lifetime (yes/no) and hazardous alcohol drinking, assessed by the Alcohol Use Disorders Identification Test (AUDIT). A score of ≥8 is considered hazardous drinking [35]. The Cronbach's alpha in our sample was α = 0.879505.

##### 2.2.1.4 Mental health measures

To evaluate depressive symptoms, we used the Patient Health Questionnaire‐9 (PHQ‐9) [40], with a total score range of 0−27. Scores of 0−4, 5−9, 10−14, 15−19 and 20−27 represent cut points for no, mild, moderate, moderately severe and severe depression, respectively. We used cut points of ≥5 for at least mild and ≥10 for at least moderate depression symptom severity [41]. The Cronbach's alpha in our sample was α = 0.90.

The Generalized Anxiety Disorder (GAD‐7) instrument was used [42], with a score range of 0−21. Scores of 0−4, 5−9, 10−14 and 15−21 represent ranges for minimal, mild, moderate and severe anxiety, respectively. We used cut points of ≥5 for at least mild and ≥10 for at least moderate anxiety symptoms severity. The Cronbach's alpha in our sample was α = 0.92.

The PTSD Checklist (PCL) was used to measure PTSD: a 17‐item self‐report questionnaire (score range 17−74) [43, 44]. A score of ≥44 is considered to be clinically relevant PTSD. The Cronbach's alpha in our sample was α = 0.96.

### 2.3 Cognitive measures

PROMIS v2.0 Adult Cognitive Function 8a short form (8‐item) was used to assess cognitive function [45]. The PROMIS v2.0 assesses the frequency of cognitive difficulties experienced in the past 7 days based on self‐report. Lower raw scores on the PROMIS v2.0 indicate greater subjective cognitive difficulty [46]. The total raw score ranges from 8 to 40. The Cronbach's alpha in our sample was α = 0.96. The 16‐item Forgetfulness Assessment Inventory was used to assess subjective memory over the past 4 weeks. A higher score corresponds to more forgetfulness or poorer memory [47]. Responses were recorded on a 5‐point Likert scale. The overall score ranges from 0 to 5 [47]. Cronbach's alpha in our sample was α = 0.95.

### 2.4 Data analysis

Descriptive statistics were used to summarize participant characteristics for history of mental illness and socio‐demographic variables. The median and interquartile range (IQR) for continuous variables were calculated for the total sample and by the self‐reported history of ever being diagnosed with any mental illness.

Percentages of participants who screened positive for mental health symptoms were calculated based on clinically relevant cut‐points of the PHQ‐9 score for depression symptoms, including at least mild (score ≥5) and at least moderate symptoms (score ≥10); cut‐points of the GAD‐7 screening instruments, for at least mild (score ≥5) and moderate (score ≥10) anxiety symptoms; and cut‐point of PCL (score ≥44) for clinically relevant PTSD.

We employed multivariable regression analyses to address two self‐reported dependent variables: ART adherence and ASES score. For each primary independent variable: clinically relevant depression symptoms, anxiety symptoms, clinically relevant PTSD, cognitive function and forgetfulness, we used logistic regression analyses to calculate crude odds ratios (OR) and 95% confidence intervals (95% CI) to evaluate associations of self‐reported ART non‐adherence: missed at least one pill in the last 2 weeks and missed at least one pill in the last week separately. For each primary independent variable, we conducted multivariable logistic regression analyses, adjusting for age, sex, education, employment, injection drug use and hazardous drinking.

The second dependent variable was the continuous ASES score. Linear regression analyses were used to calculate crude standardized regression coefficients (β) with 95% CI to predict the ASES score by each primary independent variable. Then, multivariable standardized regression coefficients (β) with 95% CI were calculated separately for each independent variable adjusted for age, gender, education, employment, injection drug use and hazardous drinking. All analyses were conducted using SAS statistical software version 9.4.

## 3 RESULTS

### 3.1 Sample characteristics

Table 1 shows the demographic, clinical and other characteristics, including adherence to ART, ASES scale, mental health symptoms, cognitive function score and forgetfulness score of the sample stratified by self‐reported mental health illness ever diagnosed in their lifetime.

**Table 1:** Socio‐demographic characteristics of the study sample stratified by self‐reported mental health diagnosis ever in lifetime

Characteristic	Overall, *N* = 230 (100%)	Mental health diagnosis ever^a^, *n* = 77 (33.5%)	No mental health diagnosis ever, *n* = 153 (66.5%)	*p*‐value
Age (mean [SD])	41.9 (10.3)	42.5 (11.3)	41.5 (10.2)	0.532
Sex				0.032
Male	136 (59.1)	38 (49.4)	98 (64.1)	
Female	94 (40.9)	39 (50.7)	55 (35.9)	
Ethnicity				0.674
Kazakh	72 (31.3)	22 (28.6)	50 (32.7)	
Russian	114 (49.6)	73 (53.3)	41 (47.7)	
Other	44 (19.6)	14 (18.2)	30 (19.6)	
Completed high education (bachelor/graduate degree)	133 (57.8)	33 (42.9)	100 (65.4)	0.001
Full‐time employment (yes/no)	180 (78.3)	60 (77.9)	120 (78.4)	0.843
Marital status				
Single (never married, divorced, separated, widowed)	114 (49.6)	37 (48.1)	77 (50.3)	0.799
Married	116 (50.4)	40 (51.9)	76 (49.7)	0.799
Injection drug use (ever)	57 (24.8)	23 (29.9)	34 (22.2)	0.205
Hazardous alcohol drinking (AUDIT score≥8)	38 (16.5)	16 (20.8)	22 (14.4)	0.217
Infectious disease history (ever in life history)	*N* (%)	*N* (%)	*N* (%)	*p*
HCV (ever had positive EIA),	74 (32.2)	27 (35.1)	47 (30.7)	0.506
HBV (ever had positive EIA)	8 (3.5)	6 (7.8)	2 (1.3)	0.018^a^
Syphilis (ever serologically positive)	29 (12.7)	11 (14.3)	18 (11.8)	0.599
Tuberculosis (ever diagnosed)	41 (17.8)	18 (23.4)	23 (15.0)	0.119
Toxoplasma (ever had positive EIA)	11 (5.0)	3 (4.0)	8 (5.5)	0.753
Cytomegalovirus (ever had positive EIA)	10 (4.4)	3 (3.9)	7 (4.7)	0.262
At least mild symptoms^*^ of depression	66 (28.7)	31 (40.3)	35 (22.9)	0.0060
At least moderate symptoms^**^ of depression	22 (9.6)	11 (14.3)	11(7.2)	0.0842
At least mild symptoms^*^ of anxiety	74 (32.2)	29 (37.7)	45 (29.4)	0.2062
At least moderate symptoms^**^ of anxiety	15 (6.5)	9 (11.7)	6 (3.9)	0.0244
At least mild symptoms^*^ of depression and anxiety	46 (20.0)	20 (26.0)	26 (17.0)	0.1081
At least moderate symptoms^**^ of depression and anxiety	13 (5.7)	7 (9.1)	6 (3.9)	0.1330^a^
Symptoms of PTSD	17 (7.4)	10 (13.0)	7(4.6)	0.0214
Self‐reported adherence (missed at least one pill of medication)				
Within the last month	42 (18.3)	12 (15.6)	30 (19.6)	0.456
Within the last 2 weeks	22 (9.6)	6 (7.8)	16 (10.5)	0.517
Within the last week	7 (3.0)	3 (3.9)	4 (2.6)	0.689^a^
Within the last 3 days	4 (1.7)	2 (1.3)	2 (2.6)	0.603^a^
	Median (IQR)	Median (IQR)	Median (IQR)	*p*
Cognitive function (raw score)	40 (34−40)	40 (32−40)	40 (35−40)	0.194
Forgetfulness score	1.44 (1.13−2.06)	1.56 (1.19−2.13)	1.44 (1.0−2.06)	0.094^b^
ASES (raw score)	120 (112−120)	120 (117−120)	120 (110−120)	0.218^b^
GAD‐7 (raw score)	2.0 (0−5)	3 (1−6)	2 (0−5)	0.009
PHQ‐9 (raw score)	2.0 (1−6)	3 (1−7)	2 (0−4)	0.004

Abbreviations: ASES, adherence self‐efficacy scale; AUDIT, alcohol use disorders identification test; EIA, enzyme immunoassay; GAD‐7, generalized anxiety disorder‐7; HBV, hepatitis B virus; HCV, hepatitis C virus; IQR, interquartile range; PHQ‐9, patient health questionnaire; PTSD, post‐traumatic stress disorder; SD, standard deviation.

^a^ Fisher.

^b^ Wilcoxon.

^c^ Diagnosed with any of the following conditions: depression, anxiety, bipolar disorder, schizophrenia or any other mental health condition.

^*^ For PHQ‐9 and GAD‐7: at least mild symptoms (score ≥ 5).

^**^ For PHQ‐9 and GAD‐7: at least moderate symptoms (score ≥ 10).

### 3.2 Associations of ART adherence with depression symptoms, anxiety symptoms, clinically relevant PTSD and cognitive function

Multivariable logistic regression analyses showed that mental health symptoms were associated with a higher probability of non‐adherence to ART (Table 2). Those who missed at least one pill in the last 2 weeks had higher odds of reporting mild (aOR = 3.34, 95% CI: 1.22–9.11, *p* < 0.05) or moderate (aOR = 4.38, 95% CI: 1.36–14.15, *p* < 0.05) depression symptoms; mild (aOR = 3.27, 95% CI: 1.20–8.92, *p* < 0.05) or moderate (aOR = 8.61, 95% CI: 2.32–31.87, *p* < 0.005) anxiety symptoms; and PTSD symptoms (aOR = 4.04, 95% CI: 1.15–14.21, *p* < 0.05) (Table 2).

**Table 2:** Unadjusted and adjusted logistic regression for participants who missed at least one pill in the last 2 weeks and in the last week symptoms of mental health symptoms, cognitive function and forgetfulness scores

	Missed at least one pill in the last 2 weeks		Missed at least one pill in the last week	
Mental health symptoms	Unadjusted OR (95% CI)	Multivariable adjusted OR (95% CI)	Unadjusted OR (95% CI)	Multivariable adjusted OR (95% CI)
Depression symptoms (at least mild, ≥5)	4.23 (1.71–10.45)^*^	3.34 (1.22–9.11)^*^	6.64 (1.26–35.13)^*^	7.74 (1.31–45.7)^*^
Depression symptoms (at least moderate, ≥10)	4.50 (1.55–13.09)^*^	4.38 (1.36–14.15)^*^	8.05 (1.68–38.67)^**^	10.05 (1.81–55.76)^**^
Anxiety symptoms (at least mild, ≥5)	3.48 (1.41–8.57)^*^	3.27 (1.20–8.92)^*^	2.92 (0.64–13.37)	4.53 (0.80–25.64)
Anxiety symptoms (at least moderate, ≥10)	8.29 (2.62–26.24)^***^	8.61(2.32–31.87)^***^	13.19 (2.65–65.71)^**^	21.33 (3.24–140.33)^***^
PTSD (≥44)	4.80 (1.51–15.25)^*^	4.04 (1.15–14.21)^*^	11.20 (2.28–55.00)^**^	13.81 (2.36–80.84)^***^
Depression and anxiety (at least moderate symptoms, ≥10)	10.77 (3.23–35.87)^***^	11.94 (2.95–48.30)^***^	15.98 (3.14–81.19)^***^	43.22 (5.28–353.54)^***^
Depression and anxiety (at least mild symptoms, ≥5)	3.98 (1.60–9.92)^*^	3.43 (1.21–9.69)^*^	5.75 (1.24–26.65)^*^	8.97 (1.61–49.91)^*^
Cognitive function raw score	0.96 (0.90–1.01)	0.97 (0.91–1.03)	0.89 (0.83–0.96)^**^	0.88 (0.81–0.96)^***^
Forgetfulness raw score	1.90 (1.13–3.19)^**^	1.60 (0.90–2.85)	3.48 (1.55–7.81)^**^	4.64 (1.76–12.24)^***^

Abbreviation: PTSD, post‐traumatic stress disorder.

^***^ *p* < 0.005; ^**^ *p* < 0.01; ^*^ *p* < 0.05.

Participants who missed at least one pill in the last week had higher odds of reporting mild (aOR = 7.74, 95% CI: 1.31–45.7, *p* < 0.05) or moderate (aOR = 10.06, 95% CI: 1.81–55.76, *p* < 0.01) depression symptoms; moderate (aOR = 21.33, 95% CI: 3.24–140.33, *p* < 0.005) anxiety symptoms and symptoms of PTSD (aOR = 13.81, 95% CI: 2.36–80.84, *p* < 0.005).

Participants with better cognitive function had lower odds of non‐adherence in the last week (aOR = 0.88, 95% CI: 0.81–0.96, *p* < 0.005). Having more forgetfulness was associated with higher odds of non‐adherence in the last week (aOR = 4.64, 95% CI: 1.76–12.24, *p* < 0.005) (Table 2).

### 3.3 Association of ART self‐efficacy with depression symptoms, anxiety symptoms, clinically relevant PTSD and cognitive function among PLWH in Almaty, Kazakhstan

Multivariable linear regression analyses showed that mental health symptoms were associated with lower adherence self‐efficacy. Those who had moderate depressive symptoms (β = −0.15, 95% CI: −0.30 to −0.01, *p* < 0.05), mild anxiety symptoms (β = −0.21, 95% CI: −0.35 to −0.07, *p* < 0.005) and moderate anxiety symptoms (β = −0.21, 95% CI: −0.34, −0.07, *p* < 0.005) and higher number of PTSD symptoms (β = −0.19, 95% CI: −0.33 to −0.05, *p* < 0.005) had lower adherence self‐efficacy scores (Table 3).

**Table 3:** Unadjusted and adjusted linear regression for ASES score of participants and symptoms of mental health symptoms, cognitive function and forgetfulness scores

Mental health symptoms	ASES score	
	Crude standardized β (95% CI)	Multivariable standardized β (95% CI)
Depression symptoms (at least mild, ≥5)	−0.14 (−0.27 to −0.00)^*^	−0.14 (−0.28 to 0.01)
Depression symptoms (at least moderate, ≥ 10)	−0.13 (−0.28 to −0.001)^*^	−0.15 (−0.30 to −0.01)^*^
Anxiety symptoms (at least mild, ≥5)	−0.21 (−0.35 to −0.08)^***^	−0.21 (−0.35 to −0.07)^***^
Anxiety symptoms (at least moderate, ≥10)	−0.19 (−0.31 to −0.06)^***^	−0.21 (−0.34 to −0.07)^***^
PTSD	−0.18 (−0.31 to −0.05)^**^	−0.19 (−0.33 to −0.05)^**^
Depression and anxiety (moderate symptoms)	−0.14 (−0.27 to −0.01)^*^	−0.16 (−0.29 to −0.02)^*^
Depression and anxiety (mild symptoms)	0.20 (−0.33 to −0.07)^***^	−0.20 (−0.35 to −0.06)^**^
Depression and PTSD	−0.17 (−0.29 to −0.03)^*^	−0.18 (−0.31 to −0.04)^*^
Anxiety and PTSD	−0.12 (−0.24 to 0.02)	−0.12 (−0.25 to 0.01)
Cognitive function score	0.26 (0.13–0.39)^***^	0.26 (0.13–0.40)^***^
Forgetfulness score	−0.27 (−0.40 to −0.14)^***^	−0.31 (−0.45 to −0.16)^***^

Abbreviations: ASES, adherence self‐efficacy scale; PTSD, post‐traumatic stress disorder.

^***^ *p* < .005; ^**^ *p* < .01; ^*^ *p* < .05.

Participants with better cognitive function had higher ASES scores (β = 0.26, 95% CI: 0.13–0.40, *p* < 0.005). A higher forgetfulness score, denoting poorer memory, was associated with a lower ASES score (β = −0.31, 95% CI: −0.45 to −0.16, *p* < 0.005) (Table 3).

## 4 DISCUSSION

This study addresses a gap in research on how mental health and cognitive function impact adherence to ART among PLWH in Kazakhstan. Our findings suggest that depression, anxiety, PTSD symptoms and worsened cognitive function are associated with poorer ART adherence and lower adherence self‐efficacy, which is consistent with previous research on PLWH in other countries [7, 8, 24−26, 48, 49, 50, 51, 52, 53].

There are multiple studies demonstrating that HIV is linked to an increased likelihood of cognitive impairment due to the underlying pathophysiology of HIV [31, 32, 33, 34, 35, 50, 54]. On the other hand, long‐term exposure to ART medications can be associated with neurocognitive impairment [55, 56]. The neurotoxic and neuropsychiatric impacts stemming from both the ART and the HIV infection itself necessitate a more comprehensive grasp of the pathological, pharmacological and behavioural mechanisms involved into the worsening of the cognitive function of PLWH [57, 58, 59, 60].

This study has several limitations. First, as a cross‐sectional study, we cannot determine the causality of relationships between mental health, cognitive function and ART adherence. Second, due to social desirability bias, participants may have overreported adherence to ART and overrated cognitive function and may have provided socially acceptable answers [61]. Third, as noted above, we did not have concurrent data on CD4^+^ cell count and HIV viral load. In addition, while T‐scores for PROMISv2, our cognitive function assessment, exist for the US general population, we did not convert our raw scores to T‐scores for these analyses since they may not be representative of PLWH in Kazakhstan. PROMISv2 T‐scores do not exist for the Kazakh population [62]. However, this study had several strengths that address some limitations in prior research including the use of a random sample from a roster of PLWH patients at the largest city AIDS Center, high participation rates and the use of validated scales of mental health and cognitive function with good reliability.

Research showed that PLWH can benefit from a wide range of mental health interventions [63]. These four models are suggested to be promising strategies for delivering efficient and effective mental healthcare in resource‐constrained settings: task shifting, stepped care, trans‐diagnostic approaches and technology‐based interventions [63, 64]. Routine screenings for mental health issues [65, 66] integrated into the HIV care continuum with mental health counselling shifting from specialized to non‐specialized health workers or trained lay persons are promising intervention approaches [67, 68, 69]. Clinicians have to be able to screen for mental health disorders and access cognitive function to refer for treatments [63] and provide support for patients with worsened cognitive function to improve their ART adherence [22, 70, 71, 72, 73, 74, 75, 76]. Technology‐based approaches including digitalized interventions that can be delivered via applications on smartphone or other devices can be effective way to support PLWH with mental illness or/and worsened cognition [77, 78].

## 5 CONCLUSIONS

The findings emphasize the vital importance of incorporating mental health and cognitive function assessments into HIV treatment programmes. This approach is essential due to the observed links between mental health issues and diminished cognitive abilities, including forgetfulness, and their correlation with reduced adherence to ART and lower self‐efficacy in treatment adherence. Strengthening mental health support and focusing on the cognitive function within these programmes can significantly enhance the adherence and overall wellbeing of PLWH.

## COMPETING INTERESTS

The authors declare no competing interests.

## AUTHORS’ CONTRIBUTIONS

GM and AD collaborated on drafting the manuscript. JD, NE‐B, LG, DG, SLR, ANP, AT and SP contributed to revisions of manuscript drafts. GM, JD, AD and ANP contributed to outlining the research objectives, sampling design and data analysis. GM and AD contributed to data analysis. JD, DG, LG, NE, AT, SP, ZN, ANP and AYD contributed substantially to the conception and design of the work and interpretation of results. AYD contributed to data collection. All authors had final approval of the version to be published and agreed to be accountable for all aspects of the work.

## FUNDING

This study was supported by the Fogarty International Center and the National Institute of Drug Abuse under Award Number D43 TW010046.PIs: Drs. Jack DeHovitz & Zhamilya Nugmanova.

## Data Availability

Data are available on request from the authors.
